# The Impact of Health Information Sharing on Hospital Costs

**DOI:** 10.3390/healthcare9070806

**Published:** 2021-06-26

**Authors:** Na-Eun Cho

**Affiliations:** College of Business, Hongik University, Seoul 04066, Korea; ncho@hongik.ac.kr

**Keywords:** health information technology, information sharing, hospital costs, poverty ratio, concentration

## Abstract

Despite substantial progress in the adoption of health information technology (IT), researchers remain uncertain as to whether IT investments benefit hospitals. This study evaluates the effect of health information sharing on the cost of care, and whether the effect varies with context. Our results suggest that information sharing using health IT, specifically the extent (breadth) and level of detail (depth) of information sharing, helps to reduce the cost of care at the hospital level. The results also show that the effects of depth of information sharing on cost savings are salient in poor and less-concentrated regions, but not in wealthier, more-concentrated areas, whereas the the effects of breadth of information sharing on cost savings are equivalent across wealth and concentration. To realize the benefits of using health IT more effectively, policy makers’ strategies for encouraging active use of health IT should be informed by market characteristics.

## 1. Introduction

More than 10 years have elapsed since the passage of the Health Information Technology for Economic and Clinical Health (HITECH) act of 2009 designed to spur adoption and promote the use of electronic health records (EHR) for the purposes of improving quality and reducing costs [1]. Putting aside ongoing debate as to whether this policy intervention has helped drive hospital adoption of health IT [2,3], the EHR adoption rate has risen substantially—more than 95% of non-federal acute care hospitals reported to possess certified health IT as of 2017 [4]. The more important question then becomes whether the use of health IT has achieved the predicted benefits.

Research to date examining how adoption of health IT affects hospital outcomes has produced mixed results regarding effects on quality and cost of care [5,6,7,8,9]. Although most studies conclude that effects are generally positive (e.g., lower morality rates, reduced costs, fewer complications, fewer unnecessary tests), some research suggests that health IT implementation does not always generate desired results [5,6,7,8,9]. EHR adoption was found by one study to have no effect on quality and costs [5], and by another to increase costs, especially in non-IT-intensive locations [7].

A number of factors could account for the inconclusive results of prior research. One is that most prior studies have focused simply on the adoption of health IT [5,6,7,8,9]. Since the federal government began emphasizing the prevalence of this new technology, determinants of and barriers to adoption, including financial, technical, psychological, social, legal, organizational, etc., have been the main subjects of the extant literature [10,11]. Although in the early stage of adoption it was difficult to collect data on use patterns, according to the information systems literature, it is use patterns—that is, how information is shared among stakeholders—not adoption, that determine the benefits an organization derives from IT [12,13,14,15]. To better understand why some institutions have realized benefits from using health IT and others have not, the present study uses data on use patterns not employed in prior studies. Further, notwithstanding previous scholars’ emphasis on the importance of taking into account context when examining the effect of health IT, research examining how the impact of health IT might vary with context remains lacking [8]. The present work addresses the mixed findings of earlier studies by examining the effects of health information sharing, specifically with regard to the extent (breadth) and level of detail (depth) of information shared, and whether the effects vary with context.

Analyzing data variously obtained from the American Hospital Association’s (AHA’s) annual and IT surveys, the Center for Medicare and Medicaid Services’ (CMS’) Hospital Compare database, and the Census Bureau’s small-area income and poverty estimates, we find that both breadth and depth of information sharing help to reduce the cost of care at the hospital level. We find depth of information sharing to provide cost savings only in poor, less-concentrated regions, not in in wealthier, more-concentrated areas, and breadth of information sharing to yield equivalent cost savings regardless of the wealth and concentration of regions. The results of the current study suggest that policy makers and practitioners carefully modify their strategies for using health IT to reflect market characteristics.

The paper is organized as follows. In Section 2, we describe our data and model, and in Section 3 discuss our empirical tests. The results are discussed in Section 4. We present our conclusions in Section 5.

## 2. Materials and Methods

### 2.1. Hypotheses Development

As mentioned in the Introduction, the present study aims to fill a gap in previous research that has produced inconclusive findings [5,6,7,8,9]. The current study examines actual use patterns, specifically, breadth and depth of information sharing, which thus far have received little attention in the literature [12]. The prior literature suggests that hospitals can realize economies of scale and achieve complementarity in operations as information is shared with more external parties [5,6]. Information sharing among multiple stakeholders can also reduce avoidable hospital readmissions and duplicate tests [16,17]. Sharing information at a detailed level reflects a degree of trust between hospitals and external parties that facilitates collaboration across these organizations [12]. This leads to the following hypothesis.

**Hypothesis** **1** (**H1**)**.** *Breadth and depth determine the degree to which information sharing decreases hospital costs.*

The current study examines differences in context that might strengthen or weaken the effect of health IT, specifically, how its effect varies with wealth and concentration in the areas in which the hospitals studied operate. We expect health IT to have a greater effect in poor (high povery ratio) than in rich (low poverty ratio) areas because the need for complementarities from other parties would be greater for those with fewer than for those with abundant resources. Similarly, we expect health IT to have a greater effect in competitive (low HHI) than in concentrated (high HHI) regions. A high concentration level indicates that a market is dominated by a small number of firms. An HHI of 1 implies that there exists only one hospital in our sample. Given only one or a few hospitals in a market, the importance of sharing information about operations decreases, reducing the marginal effect of information sharing. This leads to the following hypothesis.

**Hypothesis** **2** (**H2**)**.**  *The effects of breadth and depth of information sharing is more salient in poor, less concentrated than in wealthy, highly concentrated regions.*

### 2.2. Data

We compiled data from the American Hospital Association’s annual and IT surveys (https://www.ahadata.com/, accessed on 15 February 2021) and the Census Bureau’s small-area income and poverty estimates (https://www.census.gov/, accessed on 1 April 2021) for 2014–2016. We also obtained data on “Medicare Spending Per Beneficiary—National” for 2015–2017 from the Center for Medicare and Medicaid Services’ Hospital Compare database (https://data.cms.gov/provider-data, accessed on 1 April 2021). Note that independent and control variables are lagged by one year. We merged the data from the AHA surveys and CMS database using the respective identification numbers and added the census data using county-level FIPS codes. The AHA’s annual surveys provide data on hospital characteristics, including bed size, ownership type, teaching status, system affiliation, physician-hospital integration, revenue models, and total facility admissions. The IT surveys provide information on how broadly a hospital electronically shares patient data with other stakeholders, and the level of detail of the information that is shared. The publicly available CMS data include information on cost of care. The census data provide county-level information on poverty ratios.

### 2.3. The Model

Our main dependent variable, hospital costs, from Medicare spending per beneficiary (MSPB) at CMS Hospital Compare, is a measure of a specific hospital’s expenditure for an episode of care compared to the national median. The measure considers not only patient age and health status, but also geographic payment differences, enabling us to control patient characteristics indirectly. Note that each hospital’s expenditure is divided by the median of the national episode-weighted expenditure.

Information sharing, our main independent variable, is measured in terms of breadth and depth of information sharing. AHA IT surveys include the question, “Which of the following patient data does your hospital electronically exchange/share with one or more of the provider types listed below? (check all that apply)” We used the answers to this question to generate the variables of breadth and depth of information sharing [12]. For the breadth variable, we summed the values of the answers to the above question, (1) for hospitals within a system, (2) for hospitals outside a system, (3) for ambulatory providers within a system, and (4) for ambulatory providers outside a system. This implies that the minimum value of breadth is 0 and the maximum value is 4. For the depth variable, we summed the values of the answers to the above question, (1) for patient demographics, (2) for laboratory results, (3) for medication history, (4) for radiology reports, and (5) for clinical/summary care records in any format. This implies that the minimum value of depth is 0 and the maximum value is 5. As theorized above, we expect the coefficients of breadth and depth of information sharing to be negative.

Among several control variables included in our model, bed size, to avoid multicollinearity, is measured with 8 pre-defined codes from the AHA annual survey. Bed size ranges are (1) 6–24 beds, (2) 25–49 beds, (3) 50–99 beds, (4) 100–199 beds, (5) 200–299 beds, (6) 300–399 beds, (7) 400–499 beds, and (8) 500 or more beds. If there exist economies of scale, the ex ante expectation of the effect of bed size on hospital costs is negative; if there exist diseconomies of scale, the ex ante expectation is positive. Thus, our ex ante expectation of the effect of bed size is not predicted. For-profit ownership and government ownership are dummy variables that show differences between for-profit and government-owned hospitals, respectively. When both dummies are equal to zero, a voluntary nonprofit hospital is implied. We expect the for-profit hospital dummy to be positive, for-profit hospitals being likely to offer more profitable services, usually accompanied by expensive equipment and supplies [18]. We expect the government hospital dummy to be negative, with government-run hospitals being supported by limited funds and typically offering unprofitable services [18]. Teaching hospital is a binary variable that takes the value of 1 if the hospital is a member of the Council of Teaching Hospitals (COTH) or Association of American Medical Colleges, and 0 otherwise. We expect teaching hospital to have a negative effect on hospital costs. Contrary to the general perception that teaching hospitals are more expensive than non-teaching hospitals, it has recently been found that despite higher initial hospitalization costs, lower costs of follow-up and fewer readmissions result in overall lower total costs [19]. We also control for system affiliation and physician-hospital integration. System affiliation is a dummy variable that takes the value of 1 if a hospital is part of a system, and 0 otherwise. As with bed size, the sign of which depends on the existence of (dis)economies of scale, the ex ante expectation is not predicted. Physician–hospital integration is a binary variable that takes the value of 1 if a hospital has an arrangement (among many other arrangements) whereby physicians are employed by the hospital under an integrated salary model, and 0 otherwise. Emphasizing the integration costs that arise from changes in the behavior of physicians whose employment status changes [20], we expect the effect of physician–hospital integration on hospital costs to be positive. Capitation revenue ratio is the % of a hospital’s net revenue paid on a fixed amount per patient for delivery of healthcare services. We expect the capitation revenue ratio to have a negative effect on hospital costs, as providers with a capitated contract are encouraged to avoid unnecessary tests and procedures in order that overall costs do not exceed the fixed amount.

For market characteristics, we included as controls the poverty ratio and Herfindahl–Hirschman index (HHI). The poverty ratio is the number of people whose income falls below the poverty line divided by the total population at a county-level variable. We expect the effect of poverty ratio to be negative, people in wealthy areas being more likely to be able to afford expensive treatments. The Herfindahl–Hirschman index (HHI) is calculated based on total facility admissions. The lower the Herfindahl index, the more competitive the market. The ex ante expectation of the effect of the Herfindahl index is not predicted for the following reasons. On the one hand, hospitals in highly competitive environments are under greater pressure to strive for efficiency, thereby reducing costs. On the other hand, competition can increase costs as health insurance renders patients insensitive to prices, encouraging hospitals to provide unnecessary services [21].

$$\begin{array}{l} Hosptial\;Costs_{it+1}=\alpha +\beta_{1}Information\;Sharing_{it}\\ \;\;\;+\beta_{2}Bed\;Size_{it}+\beta_{3}For-profit_{it}+\beta_{4}Government_{it}\\ \;\;\;+\beta_{5}Teaching_{it}+\beta_{6}System\;Affiliation_{it}\\ \;\;\;+\beta_{7}Physician-Hospital\;Integration_{it}\\ \;\;\;+\beta_{8}Capitation\;Revenue\;Ratio_{it}+\beta_{9}Poverty\;Ratio_{it}\\ \;\;\;+\beta_{10}HHI_{it}+Year_{t}+\in_{it} \end{array}$$

## 3. Results

Table 1, which presents descriptive statistics for 5291 hospitals, shows the minimum hospital cost for an episode of care compared to the national median to be 0.61 and the maximum to be 1.62. In our sample, 17% are for-profit, 15% are government, and 68% are non-profit hospitals.

Table 2 shows the main results of our OLS regression analyses regarding the effect of breadth (column (1)) and depth (column (2)) of information sharing. As predicted, the coefficients of information sharing are negative and significant for both breadth and depth, supporting H1. The coefficients of for-profit ownership, teaching hospital, and capitation revenue ratio are consistent with our stated ex ante expectations, and thus not discussed further. The coefficient of bed size is positive and statistically significant, supporting the existence of diseconomies of scale. The coefficients of government ownership, system affiliation, and poverty ratio are statistically insignificant, which suggests that they do not affect hospital costs. The coefficient of physician–hospital integration is negative and statistically significant, opposite to our prediction. This result is consistent with transaction cost economics that suggest that opportunistic behavior by physicians can be controlled well within a hierarchy [22]. The coefficient of the Herfindahl index is negative and statistically significant, suggesting that competition can increase overall hospital costs.

Noting that market characteristics, the poverty ratio, and the concentration ratio are more or less deterministic from the perspective of policy makers and hospital administrators, we conducted sub-sample analyses to examine whether the effect of breadth and depth of information sharing varies across the variables: county-level poverty ratio and Herfindahl index.

In columns (1) and (2), we divide our sample into wealthy (i.e., low poverty ratio) and poor (i.e., high poverty ratio) regions by median poverty ratio. Results are reported in Table 3. Estimated coefficients of the control variables are the same for the sub-sample (Table 3) as for the full sample (Table 2) analysis. Interestingly, our results suggest that depth of information sharing reduces hospital costs only in poor (i.e., high poverty ratio) areas, as shown in column (4). The effect of depth of information sharing becomes statistically insignificant in relatively wealthy (i.e., low poverty ratio) regions, as shown in column (3). A Wald’s test confirmed that the difference in the depth coefficients across the low and high poverty ratio groups (as shown in columns (3) and (4)) is statistically significant (*p*-value < 0.1), supporting H2. Breadth of information sharing yields cost savings in both poor and wealthy areas, as shown in columns (1) and (2), not supporting H2. Overall, our results partially support H2.

We conduct an additional sub-sample analysis by dividing our full sample into more competitive (low HHI) and more concentrated (high HHI) regions by median HHI. The estimated coefficients of the control variables are the same in the sub-sample (Table 4) as in the full sample (Table 2) analysis. Our results suggest that depth of information sharing that reduces cost of care in the full sample analysis does not decrease hospital costs in concentrated areas, as shown in column (4). The coefficient of depth of information sharing is, however, negative and statistically significant in column (3), which suggests that it does decrease hospital costs in competitive regions. A Wald’s test confirmed that the difference in the depth coefficients across the low and high HHI groups is statistically significant (*p*-value < 0.1), supporting H2. Breadth of information sharing consistently decreases hospital costs regardless of concentration ratio, not supporting H2. Overall, the results from Table 4 partially support H2.

Overall, our results suggest that policy makers should consider modifying their strategies for using health IT to account for the finding that benefits are contingent on market characteristics. In countries still in an early stage of health IT investment or with limited resources, poor and competitive regions in which the benefits of health IT can be maximized should be the initial targets of implementation strategy. In countries that have already achieved nationwide adoption of EHR (e.g., the United States), the focus should be on increasing the breadth of information sharing. Policy makers should provide guidelines for increasing the level of detail of information sharing that reflect a consideration of market characteristics.

## 4. Discussion

Despite widespread adoption of EHR systems, not all hospitals seem to benefit from health IT, as evidenced by inconclusive findings regarding its effect [5,6,7,8,9]. Believing the mixed results to be a consequence of an emphasis on adoption and inattention to specific configuration strategies in information sharing, we examine how breadth and depth of information sharing affect hospital costs. There being few studies of how the effects of health IT vary with context [8], we seek to resolve the inconsistency of previous results by specifically examining different contexts (poor vs. wealthy, less concentrated and highly concentrated regions) that may intensify or weaken the effect of health IT. Our finding that depth of information sharing decreases costs in poor and competitive regions, but not in rich and concentrated regions, and that breadth of information sharing decreases overall hospital costs, has implications for both research and practice. Our study enhances the research community’s understanding of why some hospitals are successful and others unsuccessful in realizing the benefits of health IT. In the area of practice, our study provides guidance for government in promoting active use of health IT. Our findings regarding positive effects of breadth and depth of information sharing can usefully inform administrators’ and providers’ monitoring of how adopted IT systems are used. For countries with high health IT adoption to derive greater benefit from using health IT, more incentives should be given to hospitals located in poor and competitive regions. Our findings also have implications for countries that have not yet invested in EHR systems or lack the necessary resources to implement IT systems nationwide. Governments of such countries might purposefully focus on poor or competitive regions in order to maximize the effect of the limited resources they possess, these being the areas that exhibit consistent cost savings when hospitals share information either broadly or in considerable detail.

The present study’s limitations present opportunities for future research. For example, we do not have information about precise reductions in duplicate tests or treatment that can result from active information sharing among stakeholders. A future study could examine the number of CT scans or medication changes when patients receive a summary of care record electronically during the process of transitioning to another care setting. Future research could also examine the role specific information (e.g., patient demographics/laboratory results/medication history/radiology reports/clinical/summary care records) plays in reducing tests or treatment. Similarly, whereas our study considers two types of information sharing, breadth and depth, and two contexts that vary by poverty and concentration ratio, a future study might examine other types of information sharing (e.g., volume, diversity) [23] or other contexts, such as patient mix (e.g., Medicare or Medicaid share), race, specific IT vendors, etc.

## 5. Conclusions

The present research shows an understanding of health information sharing beyond mere adoption of EHR systems to be important to the realization of the benefits of health IT. The study further suggests a significant opportunity to effectively lower healthcare costs by targeting specific areas in which the effect of health IT is maximized. The results of our study can usefully guide efforts to strategically support and tailor policy to enable hospitals to achieve the overarching goal of reducing escalating healthcare costs.

## Figures and Tables

**Table 1:** Descriptive statistics.

Variable	Mean	SD	Min	Max
Hospital costs	0.985	0.074	0.610	1.620
Breadth	3.235	1.138	0	4
Depth	4.709	0.998	0	5
Bed size	4.613	1.845	1	8
For-profit	0.173	0.379	0	1
Government	0.154	0.361	0	1
Teaching	0.096	0.295	0	1
System affiliation	0.710	0.454	0	1
Physician–hospital integration	0.413	0.492	0	1
Capitation revenue ratio	0.450	0.498	0	1
Poverty ratio	15.375	5.409	3.400	46.800
HHI	0.592	0.355	0.027	1.000

**Table 2:** The impact of health information sharing on spending.

DV: Hospital Costs	(1)	DV: Hospital Costs	(2)
Breadth	−0.003 **	Depth	−0.004 **
	[0.001]		[0.002]
Bed Size	0.010 ***	Bed Size	0.010 ***
	[0.001]		[0.001]
For-profit	0.036 ***	For−profit	0.037 ***
	[0.004]		[0.004]
Government	0.002	Government	0.003
	[0.005]		[0.005]
Teaching	−0.013 ***	Teaching	−0.013 ***
	[0.004]		[0.004]
System Affiliation	−0.000	System Affiliation	−0.001
	[0.003]		[0.003]
Physician-hospital Integration	−0.008 ***	Physician−hospital Integration	−0.009 ***
	[0.003]		[0.003]
Capitation Revenue Ratio	−0.018 ***	Capitation Revenue Ratio	−0.018 ***
	[0.002]		[0.002]
Poverty Ratio	−0.000	Poverty Ratio	−0.000
	[0.000]		[0.000]
HHI	−0.048 ***	HHI	−0.048 ***
	[0.004]		[0.004]
Constant	0.984 ***	Constant	0.991 ***
	[0.009]		[0.011]
Observations	5291	Observations	5291
R-squared	0.180	R-squared	0.180

Standard errors (in brackets) are clustered at the hospital level; *** *p* < 0.01, ** *p* < 0.05.

**Table 3:** The impact of health information sharing on spending by poverty ratio.

DV: Hospital Costs	(1)	(2)	DV: Hospital Costs	(3)	(4)
	Low Poverty Ratio	High Poverty Ratio		Low Poverty Ratio	High Poverty Ratio
Breadth	−0.003 **	−0.004 ***	Depth	0.000	−0.005 ***
	[0.001]	[0.001]		[0.002]	[0.001]
Bed Size	0.013 ***	0.012 ***	Bed Size	0.000 ***	0.000 ***
	[0.001]	[0.001]		[0.000]	[0.000]
For-profit	0.042 ***	0.038 ***	For−profit	0.040 ***	0.035 ***
	[0.004]	[0.004]		[0.004]	[0.004]
Government	−0.011 ***	0.002	Government	−0.011 ***	−0.001
	[0.004]	[0.004]		[0.004]	[0.004]
Teaching	−0.005	−0.009 **	Teaching	−0.007	−0.004
	[0.005]	[0.005]		[0.006]	[0.005]
System Affiliation	0.003	0.006 *	System Affiliation	0.003	0.009 ***
	[0.003]	[0.003]		[0.003]	[0.003]
Physician-hospital Integration	−0.010 ***	−0.013 ***	Physician−hospital Integration	−0.010 ***	−0.013 ***
	[0.003]	[0.003]		[0.003]	[0.003]
Capitation Revenue Ratio	−0.014 ***	−0.015 ***	Capitation Revenue Ratio	−0.013 ***	−0.013 ***
	[0.003]	[0.003]		[0.003]	[0.003]
Constant	0.934 ***	0.939 ***	Constant	0.961 ***	0.990 ***
	[0.006]	[0.006]		[0.008]	[0.007]
Observations	2692	2599	Observations	2692	2599
R-squared	0.140	0.135	R-squared	0.110	0.101

Standard errors (in brackets) are clustered at the hospital level; *** *p* < 0.01, ** *p* < 0.05, * *p* < 0.1.

**Table 4:** The impact of health information sharing on spending by HHI.

DV: Hospital Costs	(1)	(2)	DV: Hospital Costs	(3)	(4)
	Low HHI	High HHI		Low HHI	High HHI
Breadth	−0.003 **	−0.004 ***	Depth	−0.006 ***	−0.001
	[0.001]	[0.001]		[0.001]	[0.001]
Bed Size	0.009 ***	0.012 ***	Bed Size	0.009 ***	0.012 ***
	[0.001]	[0.001]		[0.001]	[0.001]
For-profit	0.039 ***	0.035 ***	For−profit	0.038 ***	0.037 ***
	[0.004]	[0.004]		[0.004]	[0.004]
Government	−0.004	0.003	Government	−0.004	0.004
	[0.005]	[0.003]		[0.004]	[0.003]
Teaching	−0.010 **	−0.003	Teaching	−0.011 ***	−0.004
	[0.004]	[0.007]		[0.004]	[0.007]
System Affiliation	0.004	−0.001	System Affiliation	0.004	−0.002
	[0.003]	[0.003]		[0.003]	[0.003]
Physician-hospital Integration	−0.012 ***	−0.008 ***	Physician−hospital Integration	−0.012 ***	−0.008 ***
	[0.003]	[0.003]		[0.003]	[0.003]
Capitation Revenue Ratio	−0.018 ***	−0.015 ***	Capitation Revenue Ratio	−0.017 ***	−0.015 ***
	[0.003]	[0.003]		[0.003]	[0.003]
Constant	0.967 ***	0.929 ***	Constant	0.986 ***	0.925 ***
	[0.006]	[0.006]		[0.008]	[0.007]
					
Observations	2659	2632	Observations	2659	2632
R-squared	0.096	0.115	R-squared	0.101	0.112

Standard errors (in brackets) are clustered at the hospital level; *** *p* < 0.01, ** *p* < 0.05.

## Data Availability

American Hospital Association (AHA) IT survey was purchased using the fund provided by the Korea government (MIST). AHA annual survey data were provided by Chang Jongwha. The U.S. Census Bureau’s small-area income and poverty estimates that provide information about poverty ratio are publicly available (https://www.census.gov/, accessed on 1 April 2021). Hospital Compare publicly provides data on “Medicare Spending Per Beneficiary—National” where we obtained information about our dependent variable, hospital costs (https://data.cms.gov/provider-data/, accessed on 1 April 2021).
